# Risk Profile of Ischemic Stroke Caused by Small-Artery Occlusion vs. Deep Intracerebral Hemorrhage

**DOI:** 10.3389/fneur.2019.01213

**Published:** 2019-11-27

**Authors:** Zimo Chen, Jinglin Mo, Jie Xu, Haiqiang Qin, Huaguang Zheng, Yuesong Pan, Xia Meng, Jing Jing, Xianglong Xiang, Yongjun Wang

**Affiliations:** ^1^Department of Neurology, Beijing Tiantan Hospital, Capital Medical University, Beijing, China; ^2^China National Clinical Research Center for Neurological Diseases, Beijing, China; ^3^Center of Stroke, Beijing Institute for Brain Disorders, Beijing, China; ^4^Beijing Key Laboratory of Translational Medicine for Cerebrovascular Disease, Beijing, China

**Keywords:** cerebrovascular disease, lacunar stroke, intracerebral hemorrhage, risk factor, inflammation

## Abstract

**Background:** Small-artery occlusion (SAO) subtype accounts for a quarter of the cases of ischemic stroke and is mainly caused by pathological changes in cerebral small vessels, which also involve in deep intracerebral hemorrhage (dICH). However, the factors that drive some cases to SAO and others to dICH remained incompletely defined.

**Material and Methods:** This study is a cross-sectional study from the China National Stroke Registry that included consecutive patients with ischemic stroke or intracerebral hemorrhage between August 2007 and September 2008. We compared the risk profile between the two subgroups using multivariable logistic regression.

**Results:** A total of 1,135 patients with SAO stroke and 1,125 dICH patients were included for analyses. Generally, patients with SAO stroke were more likely to be male (odds ratio = 0.74, confidence interval = 0.58–0.94) and have diabetes (0.30, 0.22–0.40), higher atherogenic lipid profiles, higher body mass index (0.96, 0.94–0.99), higher waist/height ratio (0.12, 0.03–0.48), higher platelet count (0.84, 0.77–0.91), and higher proportion of abnormal estimated glomerular filtration rate (<90, ml/min/1.73 m^2^) (0.77, 0.62–0.95). Conversely, patients with dICH were more likely to have higher blood pressure parameters, inflammation levels (white blood cell count: 1.61, 1.48–1.76; high sensitivity C-reactive protein: 2.07, 1.36–3.16), and high-density lipoprotein-c (1.57, 1.25–1.98).

**Conclusions:** The risk profile between SAO stroke and dICH were different. Furthermore, despite of traditional indexes, waist/height ratio, platelet count, inflammation levels, lipid profile, and estimated glomerular filtration rate also play important roles in driving arteriolosclerosis into opposite ends.

## Introduction

Small-artery occlusion (SAO), a distinct ischemic stroke (IS) subtype resulting in small (<15 mm in axial diameter) subcortical infarcts, is thought to correlate with intrinsic disorders of perforating cerebral arterioles called arteriolosclerosis (1), due to multiple risk factors, including age, hypertension, and diabetes (2). Intracranial hemorrhage (ICH) can be classified into deep intracerebral hemorrhage (dICH) and lobar ICH, according to the location of the lesion. dICH is more likely related to longstanding hypertension (3), which results in hypertensive vasculopathy and causes microscopic degenerative changes in the wall of small-to-medium penetrating vessels (4), while lobar ICH is multimorbid and widely known as cerebral amyloid angiopathy (CAA) related disease (5, 6).

However, SAO stroke and dICH represent opposite ends of the similar pathological process in cerebral small vessels. It was shown that patients with SAO were older and more likely to have diabetes mellitus and higher cholesterol level, while dICH patients tended to be excessive alcohol consumers and have hypertension (7, 8). However, controversy still remained on other risk factors for cerebrovascular disease. It was demonstrated that renal dysfunction led to the thickening of vascular wall in arteriosclerosis (9). Moreover, previous studies have verified that platelet (PLT) also actively participate in the pathology of arteriosclerosis (10). Besides, recent findings provided strong evidence for the inflammatory hypothesis of atherosclerosis (11, 12). However, to date, no evidence has shown the above factors in differentiating SAO stroke and dICH.

Therefore, this investigation focused on more detailed differences and points of controversy between the two groups.

## Materials and Methods

### Study Cohort and Population

This is a cross-sectional study from the China National Stroke Registry (CNSR), which consecutively enrolled stroke patients (≥18 years) within 14 days after stroke onset from 132 participating hospitals in China. Stroke was defined as acute IS, intracerebral hemorrhage, and subarachnoid hemorrhage according to the World Health Organization criteria. The diagnosis was confirmed by brain computed tomography (CT) or magnetic resonance imaging (MRI) (13). The participating hospitals covered 100 tertiary and 32 secondary urban hospitals from 27 provinces and 4 municipalities in China, including the Hong Kong region, between September 2007 and August 2008. Written informed consent was issued by all participants. The study was approved by the Central Institutional Review Board at Beijing Tiantan Hospital. Demographic information (age, sex), medical variables [admission systolic blood pressure (SBP), diastolic blood pressure (DBP), pulse pressure (PP), and mean arterial pressure (MAP), reported history of hypertension, history of diabetes mellitus, history of coronary heart disease, history of dyslipidemia, history of anticoagulant drug use, and history of alcohol or tobacco use], and laboratory studies on admission [estimated glomerular filtration rate (eGFR), PLT count, white blood cell (WBC) count, high sensitivity C-reactive protein (hs-CRP) level, triglyceride (TG) level, total cholesterol (TC) level, low-density lipoprotein cholesterol (LDL-c) level, high-density lipoprotein cholesterol (HDL-c) level, waist/height ratio (WHtR), and body mass index (BMI)] were documented in paper-based case report forms.

### Defining dICH and SAO

The diagnostic criteria for SAO were defined as follows: patients who had one of the traditional clinical lacunar syndromes (including pure motor stroke, pure sensorimotor stroke, pure sensory stroke, ataxic hemiparesis, or clumsy hand dysarthria) along with brain imaging (CT or MRI) findings (infarction diameter <1.5 cm in the appropriate region) and did not show evidence of cerebral cortical dysfunction. Evidence of cardiac sources of embolism was absent, and large extracranial arteries showed a stenosis of <50% in an ipsilateral artery (14). ICH was defined according to World Health Organization criteria (13), and dICH was located at the following regions: putamen, caudate nucleus, internal capsule, thalamus, and brain stem. Cortical hemorrhage, deep intracerebral hemorrhage, intraventricular hemorrhage, prestroke modified Ranking Scale >2, lack of data for hematoma volume, and hemorrhage resulting from trauma, underlying tumor, aneurysm, or arteriovenous malformation were excluded (15).

### Risk Factor Definition

Hypertension was defined by SBP ≥ 140 mmHg and/or DBP ≥ 90 mmHg out of the acute phase or the use of pharmacological treatment for hypertension. PP was calculated as SBP–DBP, and MAP was calculated as DBP + 0.33 (SBP–DBP). Other risk factors were defined as follows: dyslipidemia (TC level ≥ 240 mg/dl, HDL-c level <35 mg/dl, or the use of lipid-lowering agents), diabetes mellitus (fasting blood glucose level ≥ 120 mg/dl or current treatment with antidiabetic drugs), current or previous smoker (an individual who smoked at the time of stroke or had quit smoking within 1 year), or heavy alcohol consumption (≥5 standard alcoholic beverages per day). We also collected information on coronary artery disease (medical history of angina, myocardial infarction, coronary artery bypass graft, or percutaneous transluminal coronary angioplasty), and treatment with anticoagulant drugs (previously documented in medical files). The details and definitions have been described previously (16).

Data were obtained from interviews with patients, next of kin, and/or attending physicians or general practitioners. Fasting lipid levels, WBC count, hs-CRP, PLT count, and eGFR measurements were carried out on venous blood samples obtained within 24 h of stroke occurrence in each participating center, using comparable procedures. eGFR was calculated using the Chronic Kidney Disease Epidemiology Collaboration creatinine equation with an adjusted coefficient of 1.1 for the Asian population (17, 18). BMI was calculated by dividing body weight in kilograms by the square of body length in meters (kg/m^2^). WHtR was obtained by dividing the waist circumference (cm) by height (cm). The atherogenic index of plasma (AIP) is defined as the base 10 logarithm of the ratio of the concentration of TG to HDL-c; the non-HDL-c is defined as TC minus HDL-c; the atherogenic index (AI) is defined as the ratio of non-HDL-c to HDL-c; and the lipoprotein combine index (LCI) is defined as the ratio of TC*TG*LDL-c to HDL-c (19).

### Statistical Analysis

Parametric continuous variables were analyzed by the *t*-test, and results were presented as means with SD. Non-parametric variables were analyzed with the Wilcoxon rank-sum test, and results are presented as medians with interquartile range. Categorical variables are presented as proportions (*n*%), and intergroup differences were tested with the χ^2^ test. Multivariable logistic regression was performed including demographic factors (age and sex) as well as a history of hypertension, SBP, DBP, history of diabetes mellitus, history of coronary heart disease, history of hypercholesterolemia, TG, TC, LDL-c, HDL-c, AIP, non-HDL-c, TC/HDL-c, LDL-c/HDL-c, AI, LCI, current or previous smoker, heavy alcohol consumption, BMI, WHtR, eGFR, PLT count, hs-CRP, and WBC count as covariates. SAO group is the reference group in evaluating odds ratios. The covariates above were analyzed by several models to avoid the selected factors concurring with each other. In model of pressure values (SBP, DBP, PP, and MAP), covariates included age, sex, history of coronary heart disease, current or previous smoker, heavy alcohol consumption, history of diabetes mellitus, TG, TC, HDL-c, BMI, PLT count, WBC count, and eGFR. In the model of traditional and nontraditional lipid profiles, covariates included age, sex, SBP, DBP, history of coronary heart disease, current or previous smoker, heavy alcohol consumption, history of diabetes mellitus, TG, TC, HDL-c, BMI, PLT count, WBC count, and eGFR. In the model of CRP, covariates included age, sex, SBP, DBP, history of coronary heart disease, current or previous smoker, heavy alcohol consumption, history of diabetes mellitus, TC, BMI, and eGFR. *P* < 0.05 on a two-sided test was considered as significant. Data were analyzed using SAS 9.4.

## Results

**Figure 1:**
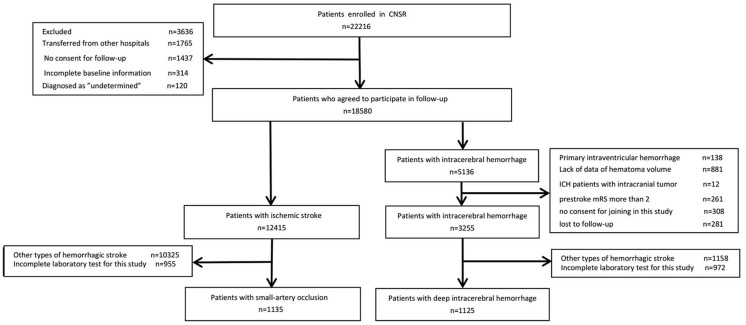
Patient flow diagram. CNSR, China National Stroke Registry; ICH, intracranial hemorrhage; mRS, modified Ranking Scale.

Of the 22,216 patients enrolled in the China National Stroke Registry, 18,580 patients had complete baseline information and agreed to participate in follow-up. Among them, 12,415 had IS and 5,136 had ICH. For IS patients, 955 without complete data of laboratory test and 10,325 for other types of IS were further excluded, leaving 1,135 SAO stroke patients included in the final analysis. For patients with ICH, after exclusion for other types of ICH, incomplete data of laboratory test and other reasons, 1,125 dICH patients were included for the comparison with SAO stroke (Figure 1).

The average age of the included patients was 63.0 ± 12.1 years (male, 63.54%). Descriptive statistics of the whole study group are summarized in Table 1. Univariable and multivariable logistic regression analyses were performed to determine the association of risk factors with stroke type (Tables 2, 3). In univariable analysis (Table 2), patients with SAO were more likely to be older, male, to smoke, to have history of diabetes, coronary heart disease, and dyslipidemia, to have higher TG, TC, LDL-c, BMI, WHtR, and PLT count and lower eGFR. Patients with dICH were more likely to be heavy alcohol consumers and have higher SBP, DBP, PP, MAP, HDL-c WBC count, and hs-CRP.

**Table 1:** Characteristics of the study population.

**Characteristics**	
Age, mean (SD), year	63.0 (12.1)
Sex, male, *n* (%)	1,436 (63.5)
History of hypertension, *n* (%)	1,546 (68.4)
Admission SBP, mean (SD), mmHg	158.4 (26)
Admission DBP, mean (SD), mmHg	92.5 (15.5)
Admission PP, mean (SD), mmHg	65.8 (19.5)
Admission MAP, mean (SD), mmHg	114.5 (17.3)
History of diabetes mellitus, *n* (%)	365 (16.2)
History of coronary heart disease, *n* (%)	38 (1.7)
History of anticoagulant drug, *n* (%)	26 (1.2)
Current smoker, *n* (%)	948 (42.0)
Heavy alcohol consumption, *n* (%)	79 (3.5)
History of dyslipidemia, *n* (%)	225 (10.0)
Admission LDL-c, median (IQR), mmol/l	2.7 (2.1–3.3)
Admission TG, median (IQR), mmol/l	1.4 (1.0–2.0)
Admission TC, median (IQR), mmol/l	4.6 (3.9–5.3)
Admission HDL-c, median (IQR), mmol/l	1.2 (1.0–1.4)
AIP, median (IQR)	0.1 −0.1–0.3)
non-HDL-C, median (IQR)	3.3 (2.7–4.0)
LDL-c/HDL-c, median (IQR)	2.2 (1.7–2.9)
TC/HDL-c, median (IQR)	3.8 (3.1–4.71)
LCI, median (IQR)	13.8 (7.4–25.6)
AI, median (IQR)	2.8 (2.1–3.7)
BMI, mean (SD), kg/m^2^	24.3 (3.9)
WHtR, mean (SD)	0.5 (0.1)
PLT count, median (IQR)	190 (149–232)
WBC count, median (IQR), 109/l	7.5 (6.1–9.5)
eGFR, median (IQR), ml/min/1.73 m^2^	83.5 (67.6–96.8)

*SBP, systolic blood pressure; DBP, diastolic blood pressure; PP, pulse pressure; MAP, mean arterial pressure; TG, triglyceride; TC, total cholesterol; LDL-c, low-density lipoprotein cholesterol; HDL-c, high-density lipoprotein cholesterol; AIP, atherogenic index of plasma; LCI, lipoprotein combine index; AI, atherogenic index; BMI, body mass index; WHtR, waist/height ratio; PLT count, platelet count; WBC count, white blood cell count; eGFR, estimated glomerular filtration rate; IQR, interquartile range; SD, standard deviation*.

**Table 2:** Univariable analysis between SAO stroke and dICH.

**Risk factors**	**Univariable Analysis**			
	**SAO stroke (*n* = 1,135)**	**dICH (*n* = 1,125)**	**OR (95% CI)**	***P*-value**
Age, mean (SD), year	64.2 (11.8)	61.7 (12.3)	0.98 (0.98–0.99)	<0.01
Sex, male, *n* (%)	757 (66.7)	679 (60.4)	0.76 (0.64–0.90)	<0.01
History of hypertension, *n* (%)	756 (66.6)	790 (70.2)	1.18 (0.99–1.41)	0.07
Admission SBP, mean (SD), mmHg	150.9 (23.3)	165.9 (26.5)	1.02 (1.02–1.03)	<0.01
Admission DBP, mean (SD), mmHg	87.7 (13.2)	97.3 (16.0)	1.05 (1.04–1.05)	<0.01
Admission PP, mean (SD), mmHg	63.2 (17.9)	68.5 (20.6)	1.01 (1.01–1.02)	<0.01
Admission MAP, mean (SD), mmHg	108.8 (15.0)	102.2 (17.6)	1.04 (1.04–1.05)	<0.01
History of diabetes mellitus, *n* (%)	227 (24.4)	88 (7.8)	0.26 (0.20–0.34)	<0.01
History of coronary heart disease, *n* (%)	29 (2.6)	9 (0.8)	0.31 (0.15–0.65)	<0.01
Current smoker, *n* (%)	504 (44.4)	444 (39.5)	0.82 (0.69–0.97)	<0.01
Alcohol consumption, *n* (%)	29 (2.6)	50 (4.4)	1.77 (1.11–2.82)	0.02
History of dyslipidemia, *n* (%)	143 (12.6)	82 (7.3)	0.55 (0.41–0.73)	<0.01
Admission LDL-c, median (IQR), mmol/l	2.8 (2.2–3.3)	2.6 (2.0–3.2)	0.83 (0.76–0.91)	<0.01
Admission TG, median (IQR), mmol/l	1.5 (1.0–2.1)	1.3 (0.9–1.7)	0.85 (0.79–0.91)	<0.01
Admission TC, median (IQR), mmol/l	4.7 (4.0–5.5)	4.5 (3.9–5.2)	0.93 (0.87–0.99)	0.03
Admission HDL-c, median (IQR), mmol/l	1.14 (0.9–1.4)	1.25 (1.0–1.5)	2.04 (1.64–2.54)	<0.01
AIP, median (IQR)	0.1 (−0.1–0.3)	0.00 (−0.2–0.2)	0.28 (0.21–0.38)	<0.01
non-HDL-c, median (IQR)	3.5 (2.8–4.2)	3.2 (2.6–3.9)	0.85 (0.80–0.92)	<0.01
LDL-c/HDL-c, median (IQR)	2.4 (1.9–3.1)	2.1 (1.6–2.7)	0.69 (0.63–0.75)	<0.01
TC/HDL-c, median (IQR)	4.1 (3.3–4.9)	3.5 (2.9–4.3)	0.77 (0.72–0.83)	<0.01
LCI, median (IQR)	16.5 (8.8–29.6)	11.3 (6.2–21.1)	0.99 (0.99–1.00)	<0.01
AI, median (IQR)	3.1 (2.3–3.9)	2.5 (1.9–3.3)	0.77 (0.72–0.83)	<0.01
BMI, mean (SD) (kg/m^2^)	24.6 (3.7)	24.1 (4.1)	0.97 (0.95–0.99)	<0.01
WHtR, mean (SD)	0.5 (0.1)	0.5 (0.1)	0.09 (0.03–0.31)	<0.01
PLT count, mean (SD), 10^12^/L	197 (3.4)	189 (2.7)		
Q1	269 (23.7)	297 (26.4)	0.92 (0.85–0.99)	0.02
Q2	281 (24.8)	297 (26.4)		
Q3	280 (24.7)	274 (24.4)		
Q4	305 (26.9)	257 (22.8)		
WBC count, median (IQR), 10^9^/L	6.9 (5.8–8.4)	8.5 (6.5–10.9)		
Q1	345 (30.4)	224 (19.9)	1.60 (1.48–1.73)	<0.01
Q2	357 (31.5)	209 (18.6)		
Q3	280 (24.7)	283 (25.2)		
Q4	153 (13.5)	409 (36.4)		
eGFR, median (IQR), ml/min/1.73 m^2^	80.7 (60.0–94.1)	85.82 (69.3–99.0)		
eGFR ≥ 90, median (IQR), ml/min/1.73 m^2^	372 (32.8)	481 (42.8)	1	
eGFR <90, median (IQR), ml/min/1.73 m^2^	763 (67.2)	644 (57.2)	0.65 (0.55–0.78)	<0.01

*SAO, small-artery occlusion; dICH, deep intracerebral hemorrhage; OR, odds ratio; CI, confidence interval; SBP, systolic blood pressure; DBP, diastolic blood pressure; PP, pulse pressure; MAP, mean arterial pressure; TG, triglyceride; TC, total cholesterol; LDL-c, low-density lipoprotein cholesterol; HDL-c, high-density lipoprotein cholesterol; AIP, atherogenic index of plasma; LCI, lipoprotein combine index; AI, atherogenic index; BMI, body mass index; WHtR, waist–height ratio; PLT count, platelet count; WBC count, white blood cell count; eGFR, estimated glomerular filtration rate; IQR, interquartile range; SD, standard deviation*.

**Table 3:** Multivariable regression analysis of indexes between SAO stroke and dICH.

**Risk factors**	**Multivariable Analysis**	***P*-value**
	**OR (95% CI)**	
Age	0.99 (0.98–1.00)	0.06
Sex, male	0.74 (0.58–0.94)	0.01
Admission SBP	1.01 (1.01–1.02)	<0.01
Admission DBP	1.03 (1.02–1.04)	<0.01
History of coronary heart disease	0.57 (0.25–1.33)	0.19
Current or previous smoker	0.74 (0.59–0.94)	0.01
Heavy alcohol consumption	1.52 (0.89–2.58)	0.12
History of diabetes mellitus	0.30 (0.22–0.40)	<0.01
Admission TG	0.89 (0.82–0.96)	<0.01
Admission TC	0.89 (0.82–0.97)	<0.01
Admission HDL-c	1.66 (1.30–2.13)	<0.01
BMI	0.96 (0.94–0.99)	<0.01
PLT count	0.84 (0.77–0.91)	<0.01
WBC count	1.61 (1.48–1.76)	<0.01
eGFR	–	
eGFR ≥ 90 ml/min/1.73 m^2^	1.00	
eGFR <90 ml/min/1.73 m^2^	0.77 (0.62–0.95)	0.02

*SAO, small-artery occlusion; dICH, deep intracerebral hemorrhage; OR, odds ratio; CI, confidence interval; SBP, systolic blood pressure; DBP, diastolic blood pressure; TG, triglyceride; TC, total cholesterol; HDL-c, high-density lipoprotein cholesterol; BMI, body mass index; PLT quartiles, platelet quartiles; WBC quartiles, white blood cell quartiles; eGFR, estimated glomerular filtration rate*.

We further performed several models of multivariable analyses in case of the factors concurring with each other. In the model adjusting medical history of hypertension, diabetes mellitus, and dyslipidemia, age, smoking, and heavy alcohol consumption were not consistently significant (Table S1). In the model adjusting indexes of pressure and serum lipid instead of the medical histories, compared with univariable analysis, age, history of coronary heart disease, and heavy alcohol consumption were not significant (Table 3). Multivariable models for MAP and PP showed that higher MAP and PP were significantly related to dICH in accord with SBP and DBP (Tables S2, S3). Moreover, in the multivariable model for WHtR instead of BMI, higher WHtR was associated with SAO, which favored the result of BMI (Table S4).

**Figure 2:**
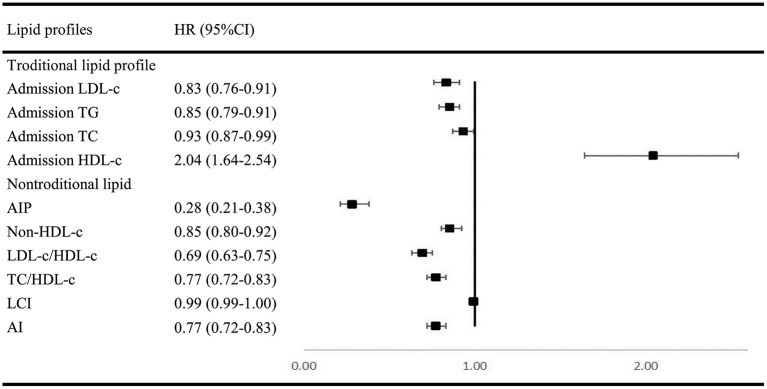
Multivariable regression analysis of lipid profile between SAO stroke and dICH. SAO, small-artery occlusion; dICH, deep intracerebral hemorrhage; OR, odds ratio; CI, confidence interval; TG, triglyceride; TC, total cholesterol; LDL-c, low-density lipoprotein cholesterol; HDL-c, high-density lipoprotein cholesterol; AIP, atherogenic index of plasma; LCI, lipoprotein combine index; AI, atherogenic index.

In multivariable analysis models for traditional and non-traditional lipid profile, all results indicated that SAO patients are likely to have higher level of atherogenic lipid profile such as higher LDL-c, TC, and TG, as well as other non-traditional lipid profile such as AIP and lower HDL-c (Figure 2).

On the contrary, higher inflammation levels were associated with higher possibility of dICH. Our results showed that dICH patients have higher WBC count than SAO patients. To consolidate the conclusion, we further performed the subgroup analysis including 449 patients with available value of hs-CRP. Consistently, we found that higher hs-CRP level was associated with dICH (Table S5).

## Discussion

The main finding of our study is that SAO stroke and dICH exhibit different risk profiles although they share similar pathophysiological process of cerebral small vessels. In comparison with previous studies, we included more detailed patient characteristics and found their significant roles in differentiating SAO stroke and dICH, such as traditional and non-traditional lipid profiles, inflammation levels, PLT count, BMI, WHtR, WBC count, hs-CRP, and eGFR in our study, which were rarely analyzed before. Thus, our work provides a more comprehensive view on the impact of risk factors in driving arteriolosclerosis into opposite ends.

In our study, history of hypertension and the specific pressure values (SBP, DBP, PP, and MAP) played a dominant role in dICH, which favored previous findings (7, 8). Contrary to hypertension, diabetes mellitus was associated with SAO stroke, which was also consistent with previous reports (19–21). Furthermore, we observed that elevated PLT count were more strongly associated with SAO stroke. Platelets play a key role in the development of IS via their role in evolution of atherosclerosis (22). In contrast, thrombocytopenia has been found to be associated with spontaneous intracerebral hemorrhage (23). In addition, PLT count have also been recorded as an important index for development and prognosis of ischemic and hemorrhagic stroke (24, 25).

Investigations remain limited by far on BMI or WHtR between SAO and dICH. In accordance with previous studies (26–29), our findings agreed that lower BMI and WHtR were more relevant to dICH. We hypothesize that the adipose tissue may play an important role in shifting the cerebral small vessel disease manifestations toward either ischemia or hemorrhage (28). Furthermore, we compared the role of various blood lipid parameters including traditional (TG, TC, LDL-c, and HDL-c) and non-traditional lipid profiles (AIP, non-HDL-c, TC/HDL-c, LDL/HDL-c, AI, and LCI) between dICH and SAO stroke. Numerous studies focused on the association between lipid profiles and stroke subtypes but yielded conflict results. The China Kadoorie Biobank study with 512,891 participants found that plasma concentrations of LDL-C and TG were positively associated with risk of IS and inversely associated with a risk of ICH. Moreover, plasma concentrations of HDL-C were inversely associated with risk of IS, but not with ICH. Furthermore, the causal relevance of LDL-C for both IS and ICH was confirmed by Mendelian randomization analyses in this study (30). However, two studies enrolled elderly adults and observed no association between LDL-C levels and the risk of ICH (31, 32). In Women's Health Study with 27,937 women enrolled, they reported that low LDL-C levels and low TG levels were associated with increased risk of hemorrhagic stroke, but no association between HDL-C levels and the risk of ICH was found. Most lipid profiles analyzed in our study were shown to significantly participate in differentiating SAO from dICH (33, 34). The analyses demonstrated that ICH patients tend to have lower level of atherogenic lipid profiles. It is supported by the histopathological studies demonstrating that lower cholesterol concentrations may increase permeability of the vessel walls (35, 36), causing arterionecrosis and microaneurysms, which is often found in ICH. AIP was shown to be a better marker to reflect increased cardiovascular disease risk than TC, LDL-c, HDL-c, and other nontraditional lipid profiles (37). Our results also showed that AIP may have a better capability in distinguishing SAO from dICH.

Evidence suggested that inflammation played a key role in the pathogenesis of cardiovascular diseases (38), and increased inflammation levels were related to the severity, disability, and mortality of both ischemic and hemorrhagic stroke (39). However, it was rarely considered in analysis as a differential characteristic between dICH and SAO stroke. Previous studies have shown that inflammatory cytokines were involved in the pathogenesis of cerebral small vessel disease and differing inflammatory pathways between ischemic and hemorrhagic manifestations of cerebral small vessel disease (40). As markers of inflammation, the increase in WBC count and hs-CRP was significantly associated with dICH compared with SAO group in our investigation. Thus, we proposed that the intensive systematic inflammation may involve in the weakening of vessel wall and lead to vessel rupture. However, due to the design of this cross-sectional study, it remains unknown whether the observed higher level of hs-CRP in ICH patients results from the inflammation or is just an acute phase reactant.

Our study has several limitations: First, the lack of MRI data about the presence/absence of neuroradiological markers of small vessel disease prevented us from further revealing the differences such as white matter hyperintensities and microbleed between SAO stroke and dICH, and may cause the confounders for selection of cases for the present study. Second, our study is a cross-sectional study, but the indexes such as blood pressure, lipid values, and WBC count were collected at the acute phase of stroke, which cannot fully reflect the long-term behavior. Third, because of the lack of a stroke-free control group, we cannot conclude but speculate that the observed associations might represent the causality in a way, and further relationships need to be investigated in the future. Fourth, the lack of centralization for biological measures and radiological data may also cause the potential biases. Fifth, our study population is based on Asian population; therefore, the findings may not be generalized directly to all populations. Our study needs to be investigated in a longitudinal prospective cohort to further reveal the association between risk factors and different subtype of stroke occurrence. In addition, elucidating the differences in neuroradiological markers of small vessel disease by MRI is needed to deeply demonstrate the underlying mechanism between SAO and dICH in future investigation.

## Conclusions

On the basis of the traditional risk profiles, this study adds evidence that higher PLT count, higher level of atherogenic lipid profile, and abnormal eGFR were in favor of SAO stroke as compared to dICH. Patients with dICH were more likely to have higher inflammation levels and higher HDL-c.

## Data Availability Statement

The datasets generated for this study are available on request to the corresponding author.

## Ethics Statement

The studies involving human participants were reviewed and approved by the ethics committee of Beijing Tiantan Hospital. The patients/participants provided their written informed consent to participate in this study.

## Author Contributions

YW contributed to the conception and design of the study. ZC and JM contributed to manuscript drafting and critical revisions of the manuscript. JX, HQ, HZ, XM, and JJ contributed to the acquisition and analysis of data. YP and XX contributed to statistical analysis.

### Conflict of Interest

The authors declare that the research was conducted in the absence of any commercial or financial relationships that could be construed as a potential conflict of interest.
