# Supplementary material for: Risk Profile of Ischemic Stroke Caused by Small-Artery Occlusion vs. Deep Intracerebral Hemorrhage
Source: Front Neurol. 2019 Nov 27;10:1213. doi: 10.3389/fneur.2019.01213 (PMC6890715; doi:10.3389/fneur.2019.01213)
Supplement: Supplementary file 1 [file Table_1.DOCX]

**Supplementary material**

**Table S1.** Multivariable regression analysis of medical history between SAO stroke and dICH.

| Risk factors | Multivariable analysis | *P* value |
| --- | --- | --- |
|  | OR (95% CI) |  |
| Age | 0.99 (0.98-1.00) | 0.05 |
| Sex, male | 0.80 (0.64-0.99) | 0.04 |
| History of hypertension | 1.37 (1.14-1.66) | <0.01 |
| History of diabetes mellitus | 0.28 (0.21-0.36) | <0.01 |
| History of coronary heart disease | 0.38 (0.17-0.83) | 0.02 |
| Current or previous smoker | 0.81 (0.65-1.00) | 0.05 |
| Alcohol consumption | 1.60 (0.99-2.58) | 0.06 |
| History of dyslipidemia | 0.64 (0.47-0.87) | <0.01 |
| BMI | 0.97(0.95-0.99) | 0.01 |
| eGFR | ---- |  |
| eGFR ≥ 90 ml/min/1.73m^2^ | 1.00 |  |
| eGFR < 90 ml/min/1.73m^2^ | 0.74 (0.61-0.91) | <0.01 |

Abbreviations: SAO, small-artery occlusion; dICH, deep intracerebral hemorrhage; OR, odds ratio; CI, confidence interval; BMI, body mass index; eGFR, estimated glomerular filtration rate.

**Table S2.** Multivariable regression analysis between SAO stroke and dICH for PP.

| Risk factors | Multivariable regression analysis | *P* value |
| --- | --- | --- |
|  | OR (95% CI) |  |
| Age | 0.98 (0.97-0.99) | <0.01 |
| Sex, male | 0.82 (0.65-1.03) | 0.08 |
| Admission PP | 1.02 (1.01-1.02) | <0.01 |
| History of coronary heart disease | 0.49 (0.22-1.10) | 0.08 |
| Current or previous smoker | 0.75 (0.60-0.94) | 0.01 |
| Heavy alcohol consumption | 1.45 (0.87-2.41) | 0.16 |
| History of diabetes mellitus | 0.28(0.21-0.37) | <0.01 |
| Admission TG | 0.89 (0.83-0.96) | <0.01 |
| Admission TC | 0.90 (0.83-0.98) | 0.01 |
| Admission HDL-c | 1.76 (1.38-2.25) | <0.01 |
| BMI | 0.98 (0.96-1.00) | 0.08 |
| PLT count | 0.82 (0.76-0.90) | <0.01 |
| WBC count | 1.66 (1.53-1.81) | <0.01 |
| eGFR | ---- |  |
| eGFR ≥ 90 ml/min/1.73m^2^ | 1.00 |  |
| eGFR < 90 ml/min/1.73m^2^ | 0.81 (0.66-0.99) | 0.04 |

Abbreviations: SAO, small-artery occlusion; dICH, deep intracerebral hemorrhage; OR, odds ratio; CI, confidence interval; PP, pulse pressure; TG, triglyceride; TC, total cholesterol; HDL-c, high-density lipoprotein cholesterol; BMI, body mass index; PLT count, platelet counts; WBC count, white blood cell count; eGFR, estimated glomerular filtration rate.

**Table S3.** Multivariable regression analysis between SAO stroke and dICH for MAP.

| Risk factors | Multivariable regression analysis | *P* value |
| --- | --- | --- |
|  | OR (95% CI) |  |
| Age | 0.99 (0.98-1.00) | 0.05 |
| Sex, male | 0.74 (0.58-0.94) | 0.01 |
| Admission MAP | 1.04 (1.04-1.05) | <0.01 |
| History of coronary heart disease | 0.57 (0.25-1.32) | 0.19 |
| Current or previous smoker | 0.74 (0.59-0.94) | 0.01 |
| Heavy alcohol consumption | 1.51 (0.89-2.57) | 0.13 |
| History of diabetes mellitus | 0.30 (0.22-0.40) | <0.01 |
| Admission TG | 0.89 (0.82-0.96) | <0.01 |
| Admission TC | 0.89 (0.81-0.97) | <0.01 |
| Admission HDL-c | 1.66 (1.30-2.13) | <0.01 |
| BMI | 0.96 (0.94-0.99) | <0.01 |
| PLT count | 0.84 (0.77-0.92) | <0.01 |
| WBC count | 1.61 (1.48-1.76) | <0.01 |
| eGFR | ---- |  |
| eGFR ≥ 90 ml/min/1.73m^2^ | 1.00 |  |
| eGFR < 90 ml/min/1.73m^2^ | 0.77 (0.62-0.95) | 0.02 |

Abbreviations: SAO, small-artery occlusion; dICH, deep intracerebral hemorrhage; OR, odds ratio; CI, confidence interval; MAP, mean arterial pressure; TG, triglyceride; TC, total cholesterol; HDL-c, high-density lipoprotein cholesterol; BMI, body mass index; PLT count, platelet count; WBC count, white blood cell count; eGFR, estimated glomerular filtration rate.

**Table S4.** Multivariable regression analysis between SAO stroke and dICH for WHtR.

| Risk factors | Multivariable analysis | *P* value |
| --- | --- | --- |
|  | OR (95% CI) |  |
| Age | 0.99 (0.98-1.00) | 0.09 |
| Sex, male | 0.72 (0.57-0.92) | <0.01 |
| Admission SBP | 1.01 (1.01-1.02) | <0.01 |
| Admission DBP | 1.03 (1.02-1.04) | <0.01 |
| History of coronary heart disease | 0.58 (0.25-1.33) | 0.18 |
| Current or previous smoker | 0.75 (0.60-0.95) | 0.02 |
| Heavy alcohol consumption | 1.46 (0.86-2.49) | 0.14 |
| History of diabetes mellitus | 0.30 (0.23-0.41) | <0.01 |
| Admission TG | 0.89 (0.82-0.96) | <0.01 |
| Admission TC | 0.88 (0.81-0.96) | <0.01 |
| Admission HDL-c | 1.70 (1.32-2.18) | <0.01 |
| WHtR | 0.12 (0.03-0.48) | <0.01 |
| PLT count | 0.84 (0.77-0.91) | <0.01 |
| WBC count | 1.61 (1.48-1.76) | <0.01 |
| eGFR | ---- |  |
| eGFR ≥ 90 ml/min/1.73m^2^ | 1.00 |  |
| eGFR < 90 ml/min/1.73m^2^ | 0.77 (0.62-0.95) | 0.01 |

Abbreviations: SAO, small-artery occlusion; dICH, deep intracerebral hemorrhage; OR, odds ratio; CI, confidence interval; SBP, systolic blood pressure; DBP, diastolic blood pressure; TG, triglyceride; TC, total cholesterol; HDL-c, high-density lipoprotein cholesterol; WHtR, waist–height ratio; PLT count, platelet count; WBC count, white blood cell count; eGFR, estimated glomerular filtration rate.

**Table e-5.** Multivariable regression analysis between SAO stroke and dICH for CRP.

| Risk factors | SAO stroke  (n = 233) | dICH  (n = 216) | Univariate analysis  OR (95% CI) | Multivariable analysis  OR (95% CI) | *P* value for Multivariable analysis |
| --- | --- | --- | --- | --- | --- |
| Age, mean (SD), y | 64.5 (12.3) | 64.5 (12.3) | 0.99 (0.97-1.00) | 1.00 (0.98-1.02) | 0.99 |
| Sex, male, n (%) | 115 (66.5) | 129 (59.7) | 0.75 (0.51-1.10) | 0.57 (0.34-0.96) | 0.03 |
| Admission SBP, mean (SD), mm Hg | 151.4 (21.6) | 164.6 (26.7) | 1.02 (1.02-1.03) | 1.01 (1.00-1.02) | 0.18 |
| Admission DBP, mean (SD), mm Hg | 86.8 (13.1) | 95.9 (15.8) | 1.05 (1.03-1.06) | 1.03 (1.01-1.06) | <0.01 |
| History of coronary heart disease, n (%) | 6 (2.6) | 4 (1.9) | 0.71 (0.20-2.57) | 1.15 (0.28-4.65) | 0.84 |
| Current or previous smoker, n (%) | 97 (41.63) | 92 (42.59) | 1.04 (0.72-1.51) | 1.05 (0.64-1.72) | 0.86 |
| Heavy alcohol consumption, n (%) | 2 (0.86) | 13 (6.02) | 7.39 (1.65-33.12) | 9.03 (1.89-43.14) | <0.01 |
| History of diabetes mellitus, n (%) | 60 (25.8) | 22 (10.2) | 0.33 (0.19-0.56) | 0.46 (0.26-0.81) | <0.01 |
| Admission TC, median (IQR), mmol/L | 4.7 (4.0-5.5) | 4.7 (3.9-5.3) | 0.88 (0.75-1.04) | 0.86 (0.71-1.04) | 0.11 |
| BMI, mean (SD) (kg/m^2^) | 24.6 (4.2) | 24.5 (3.7) | 1.00 (0.95-1.05) | 0.99 (0.94-1.04) | 0.59 |
| eGFR median (IQR), ml/min/1.73m^2^ | 80.8 (63.6-94.8) | 87.4 (71.1-98.3) |  | ---- |  |
| eGFR ≥ 90 ml/min/1.73m^2^ | 79 (33.9) | 95 (44.0) | 1.00 | 1.00 |  |
| eGFR < 90 ml/min/1.73m^2^ | 154 (66.1) | 121 (56.0) | 0.65 (0.45-0.96) | 0.68 (0.42-1.12) | 0.13 |
| CRP | 1.68 (0.5-4.6) | 3.17 (0.5-8.6) | 1.95 (1.34-2.84) | 2.07 (1.36-3.16) | <0.01 |

Abbreviations: SAO, small-artery occlusion; dICH, deep intracerebral hemorrhage; OR, odds ratio; CI, confidence interval; SBP, systolic blood pressure; DBP, diastolic blood pressure; TC, total cholesterol; BMI, body mass index; eGFR, estimated glomerular filtration rate; IQR, interquartile range; SD, standard deviation.
